# Ultrasonication-derived CAR-T vesicles preserve antigen-specific cytotoxic function

**DOI:** 10.3389/fonc.2026.1895812

**Published:** 2026-08-14

**Authors:** E.A. Zmievskaya, I.A. Ganeeva, A.M. Rogov, S. Spada, E.R. Bulatov

**Affiliations:** 1 Institute of Fundamental Medicine and Biology, Kazan Federal University, Kazan, Russia; 2 Tumor Immunology and Immunotherapy Unit, Scientific Institute for Research, Hospitalization and Healthcare (IRCCS)-Regina Elena National Cancer Institute, Rome, Italy; 3 Shemyakin-Ovchinnikov Institute of Bioorganic Chemistry, Russian Academy of Sciences, Moscow, Russia

**Keywords:** antigen-specific cytotoxicity, apoptosis, artificial vesicles, CAR-T cells, cell-free immunotherapy, extracellular vesicles, solid tumors, ultrasonication

## Abstract

**Introduction:**

Chimeric antigen receptor (CAR) T-cell therapy has transformed the treatment of hematologic malignancies but remains largely ineffective against solid tumors, where stromal barriers and an immunosuppressive microenvironment restrict T-cell infiltration and function. Cell-free vesicular derivatives of therapeutic lymphocytes may help overcome these limitations while preserving antigen specificity.

**Methods:**

Artificial vesicles were generated from CAR-T cells (CAR-AVs) by brief ultrasonication followed by differential centrifugation. Their physicochemical and functional properties were evaluated in vitro using scanning electron microscopy, flow cytometry with DiO labeling and recombinant CD19–PE, real-time impedance analysis, and qRT-PCR of apoptosis-associated genes.

**Results:**

Scanning electron microscopy revealed nanoscale, membrane-enclosed vesicles with mean ± SD diameters of 132 ± 76 nm for CAR-AVs and 149 ± 63 nm for T cell-derived vesicles (T-AVs). Flow cytometry detected CAR expression on 52.7 ± 20.5% of CAR-AVs, compared with 8.3 ± 6.1% background staining in T-AVs and 64.2 ± 14.4% CD19–PE-positive parental CAR-T cells. At a protein-normalized dose of 15 µg mL–1, CAR-AVs selectively suppressed the proliferation of CD19-expressing tumor cells, reducing the cell index by approximately 26–27% in PC3M(CD19+) cultures and 68% in A431(CD19+) cultures, with minimal effects on antigen-negative counterparts. T-AVs induced only modest, antigen-independent suppression. In antigen-positive models, CAR-AV treatment upregulated *MDM2, CDKN1A/p21, BBC3/PUMA*, and *BAX*, consistent with activation of stress- and apoptosis-associated signaling pathways.

**Discussion:**

Ultrasonication-derived CAR-AVs preserve detectable CAR display and retain antigen-associated antitumor activity in solid-tumor models. These findings support CAR-AVs as a promising and potentially scalable cell-free complement to CAR-T therapy, while highlighting the need for particle-resolved quantification, dose-response studies, and *in vivo* validation.

## Introduction

Chimeric antigen receptor (CAR) T cell therapy has revolutionized the treatment of CD19^+^ and BCMA^+^ hematologic malignancies, yet its efficacy in solid tumors remains limited (1). Two interrelated barriers largely account for this reduced performance: (i) inefficient trafficking and infiltration of T cells through the dense, fibroinflammatory stroma characteristic of solid tumors, and (ii) the profoundly immunosuppressive tumor microenvironment (TME), which imposes checkpoint inhibition, metabolic restriction, and cytokine-mediated suppression of T cell activity; (2). Overcoming these limitations will require alternative therapeutic modalities capable of preserving the targeting precision of CAR-T cells while bypassing the structural and immunologic constraints imposed by the TME.

Extracellular vesicles (EVs), membrane-bound and non-replicating particles that encapsulate proteomic and nucleic acid signatures of their parental cells, have emerged as promising vehicles for cell-free immunotherapy (3). EVs secreted by CAR-T cells (historically referred to as “exosomes” within the small-EV fraction) have been shown to recapitulate the antigen-specific cytotoxic activity of their parent cells while lacking inhibitory receptors such as PD-1, making them less susceptible to PD-L1–mediated immune evasion. In addition, preclinical studies suggest that CAR-T–derived EVs exhibit attenuated cytokine-release profiles and may therefore provide a more favorable safety margin than live-cell therapies (4, 5). Another advantage of this platform is its intrinsic safety, as vesicles are unable to proliferate, unlike living cells. Furthermore, their functional properties may potentially be modulated by loading additional cargo, including proteins, nucleic acids, or chemotherapeutic agents (6). At the same time, CAR-T-derived exosomes face important challenges, particularly the scalability and standardization of manufacturing technologies, which remain major obstacles to clinical translation (7).

Despite this promise, manufacturing remains the principal bottleneck for EV-based therapeutics. Effective doses in preclinical studies typically reach the milligram-per-kilogram range (≈10^12^ particles/kg), whereas clinical investigations often employ repeated administrations of only 10^8–^10^9^ particles or several hundred micrograms of EV protein (8). Given that a single T lymphocyte secretes only 50–100 EVs, achieving therapeutic yields through passive secretion alone would require >10^10^ parent cells per kilogram, a scale that is impractical for routine clinical manufacturing (9). To address this problem, various biological stimulation and stress-induced secretion strategies have been explored, including hypoxia, nutrient deprivation, and mechanical stimuli, but these approaches have yielded only incremental improvements in EV output (10).

A conceptually distinct and increasingly attractive strategy is the top-down generation of cell-derived nanovesicles, also referred to as artificial vesicles (AVs). These particles are produced by physical fragmentation of cellular membranes into EV-sized structures, enabling the rapid generation of large amounts of bioactive vesicles. Among the available methods, ultrasonication offers particular advantages due to its speed, scalability, and accessibility, and it has been shown to generate functionally active vesicles from diverse immune cell types, including regulatory T cells (11, 12). However, it has not been rigorously established whether vesicles generated from CAR-T cells by ultrasonication retain surface CAR display and preserve antigen-restricted effector function.

In this study, we address this question using a CD19-directed CAR-T model and MISEV-compliant terminology and experimental standards. We report the generation of CAR-T–derived artificial vesicles (CAR-AVs) by brief ultrasonication and assess their structural, phenotypic, and functional characteristics relative to vesicles from unmodified T cells (T-AVs) and parental cells. Using two human solid-tumor models engineered to express CD19 (PC3M and A431), we test the hypothesis that CAR-AVs preserve membrane-displayed CAR molecules and exert antigen-dependent cytostatic and cytotoxic effects, as reflected by real-time proliferation dynamics and activation of apoptosis-related gene programs.

By combining a scalable vesicle manufacturing strategy with functional validation, this work establishes a feasible cell-free extension of CAR-T logic and positions ultrasonication-derived CAR-AVs as a manufacturable and translationally relevant platform for the treatment of solid tumors. Such an approach has a potential to overcome mentioned obstacles in CAR-T therapy of solid tumors and broaden its application and efficacy.

## Materials and methods

### Generation of CD19 CAR-T cells

A previously established second-generation CD19-specific chimeric antigen receptor (CAR) construct (13) was employed. Lentiviral particles were generated in HEK293T cells using a three-plasmid packaging system (CAR transfer vector: psPAX2: pMD2.G, 3:3:1 mass ratio). Transfection was performed with PEI MAX (Polysciences, USA) at a total plasmid DNA concentration of 1.5 µg/mL culture medium.

Peripheral blood mononuclear cells (PBMCs) were isolated from healthy donor blood via density-gradient centrifugation on Ficoll (1.077 g/mL; Paneko, Russia) at 800 × g for 30 min. The buffy coat was collected, washed twice in Dulbecco’s phosphate-buffered saline (DPBS), and resuspended in RPMI-1640 medium (Paneko, Russia) supplemented with L-glutamine, penicillin–streptomycin (100 U/mL and 100 μg/mL respectively), and 10% fetal bovine serum (FBS; Biosera, France) at 2 × 10^6^ cells/mL. T cells were activated for 48 h using T Cell TransAct (Miltenyi, USA) following the manufacturer’s protocol, in the presence of 300 IU/mL IL-2 (Prospec, Israel).

After 48 h of activation, 1 × 10^6^ viable T cells were washed with DPBS and resuspended in RPMI-1640 containing the CAR lentiviral vector (MOI 10), TransAct, IL-2 (300 IU/mL), and protamine sulfate (20 µg/mL; Sigma-Aldrich, USA). Transduction was carried out by spinoculation at 1500 × g for 2 h at 32 °C, followed by overnight incubation (16 h). Cells were then washed with DPBS and maintained in complete medium supplemented with IL-2 (≤ 300 IU/mL) until use. Activated but non-transduced T cells were cultured in parallel and served as negative controls.

### Production of artificial vesicles

Artificial vesicles (AVs) were produced from both CAR-T cells (CAR-AVs) and activated, untransduced T cells (T-AVs) by ultrasonication. Cells were washed twice in DPBS and resuspended in serum-free medium. Ultrasonication was performed on ice using a Qsonica Q125 homogenizer (Qsonica, USA) operating at 20 kHz and 30% amplitude (255 µm), with 1 s on/1 s off pulses for a total sonication duration of 1 min. The sonication regimen was selected on the basis of our previous empirical work with primary T lymphocytes as a compromise between vesicle yield and preservation of vesicle integrity (11). Pulsed sonication on ice was used to limit sample heating and excessive mechanical stress, which could otherwise affect particle size distribution and reduce retention of membrane proteins, including CAR molecules.

Vesicles were purified through differential centrifugation: 300 × g for 10 min to remove intact cells, followed by 2000 × g for 10 min to eliminate debris and apoptotic bodies. The vesicle-containing fraction was pelleted at 15,000 × g for 20 min, washed twice with DPBS under identical conditions, and resuspended in DPBS or culture medium as required. This protocol enriches particles primarily within the ~100–1000 nm range, with smaller vesicles remaining in the supernatant. Total protein concentration was quantified by BCA assay (Pierce BCA, Thermo Fisher Scientific, USA) and used for vesicle dosage normalization.

### Scanning electron microscopy

For ultrastructural analysis, AVs were washed twice (15,000 × g, 20 min) and resuspended in 0.1 M sodium phosphate buffer (pH 7.4; 0.018 M NaH_2_PO_4_, 0.081 M Na_2_HPO_4_). Vesicles were deposited onto defatted glass slides by centrifugation at 700 × g for 1 h in six-well plates. Samples were fixed with 10% glutaraldehyde, rinsed with ultrapure water, and dehydrated through a graded ethanol series (30%, 50%, 70%, 80%, and 96%; 15 min each, with the 96% step repeated twice).

Air-dried samples were coated with a 15 nm Au/Pd conductive layer (80/20) using a Q150T ES sputter system (Quorum Technologies, UK) and imaged on a Merlin scanning electron microscope (Carl Zeiss, Germany) at 5 kV and 300 pA probe current. Sixteen independent fields per sample were acquired, and vesicle diameters were quantified using ImageJ software.

### Flow cytometry

For cellular staining, 3 × 10^5^ T cells or CAR-T cells were washed twice with DPBS (400 × g, 5 min), resuspended in 50 µL DPBS, and incubated for 20 min with recombinant CD19–phycoerythrin (CD19-PE) (Sino Biological, China, HR12NO2201). After two additional washes with DPBS, the samples were resuspended in 300 µL DPBS and analyzed by flow cytometry. T cells and CAR-T cells were analyzed separately from AV samples using a conventional flow cytometry protocol with gating defined relative to unstained cell controls.

For vesicle staining, AVs were washed (15,000 × g, 20 min), resuspended in 600 µL DPBS, and labeled with the lipophilic membrane dye DiO (Thermo Fisher, USA, V22886) for 15 min. Following centrifugation and washing, DiO-labeled vesicles were stained with recombinant CD19-PE as described above. Flow cytometry was performed using a BD FACSAria III (BD Biosciences, USA), with at least 10,000 events acquired per sample. DiO-positive events were gated as vesicles, and within this population CD19-PE positivity was used to determine the fraction of CAR-bearing vesicles.

According to this approach, vesicles are identified using dual labeling with a membrane dye (DiO) and a labeled protein ligand (CD19-PE) recognizing the CAR molecule. The fluorescence threshold (zero gate) was defined using an unstained vesicle sample. This strategy helps distinguish vesicular events from non-vesicular particles such as protein aggregates or micelles. Because very small particles scatter light weakly in the forward direction, fluorescence signals were analyzed relative to side scatter (SSC) rather than forward scatter (FSC). The instrument was regularly calibrated using BD FACSDiva™ CS&T Research Beads (BD Biosciences, USA) to ensure consistent performance. (14).

### Cell culture

Human tumor cell lines PC3M, PC3M(CD19^+^), A431, and A431(CD19^+^) were cultured in Dulbecco’s Modified Eagle Medium (DMEM) (Paneco, Russia) supplemented with 5% FBS (Biosera, France). Cells were maintained at 37 °C in a humidified incubator with 5% CO_2_ and passaged at ~80% confluency.

### Real-time cell analysis

The effects of AVs on tumor cell proliferation were assessed using the xCELLigence Real-Time Cell Analysis (RTCA) system (ACEA Biosciences, USA). Tumor cells [PC3M, PC3M(CD19^+^), A431, A431(CD19^+^)] were seeded into E-Plate 16 wells (5 × 10³ cells/well, 150 µL medium) and allowed to adhere for 24 h.

AVs were added at a final concentration of 15 µg/mL (total protein). As cellular controls, T cells or CAR-T cells were co-cultured at an effector: target (E:T) ratio of 1:1. The cell index (CI) was recorded every 15 min, normalized to the last time point before AV or cell addition, and analyzed over time to assess cytostatic and cytotoxic effects.

### Quantitative real-time PCR

To quantify pro-apoptotic gene expression, tumor cells were seeded into six-well plates (2 × 10^5^ cells/well in 1 mL medium) and incubated for 24 h. Cells were then treated with AVs (15 µg/mL total protein) for 72 h. Following incubation, cells were detached with trypsin, washed twice with DPBS, and total RNA was extracted using PureZol reagent (Bio-Rad, USA).

Complementary DNA (cDNA) was synthesized using M-MLV reverse transcriptase (Eurogen, Russia) and Random(dN)_10_ primers (Eurogen, Russia). qRT-PCR was performed with 5× qPCRmix-HS (Eurogen, Russia). Primer sequences for *TP53*, *MDM2*, *CDKN1A (p21)*, *BBC3 (PUMA)*, *BAX*, and *ACTB* are listed in **Table 1**. Expression levels were normalized to *ACTB* as the reference gene.

**Table 1:** Primer and probe sequences used for RT-qPCR quantification of target and reference genes.

Target gene	Sequence
*TP53*	Forward: GCCATCTACAAGCAGTCACAG Reverse: TCATCCAAATACTCCACACGC Probe: [HEX]CCCCTCCTCAGCATCTTATCCGAGT[BH2]
*CDKN1A(p21)*	Forward: GCCTCCTCATCCCGTGTTCT Reverse: GTACCACCCAGCGGACAAGT Probe: [HEX]AGCCGGCCCACCCAACCTCCG[BHQ2]
*MDM2*	Forward: TGTGCAAAGAAGCTAAAGAAAAGG Reverse: AGGTTGTCTAAATTCCTAGGGTTAT Probe: [HEX]ATTGGTTGTCTACATACTGGGCAGGG[BHQ2]
*PUMA (BBC3)*	Forward: GGGCCCGTGAAGAGCAAATG Reverse: CTGGCTCAGGGAAGATGGCT Probe: [FAM]CGGTTGCTCCAGCCCGGCGC[BHQ1]
*BAX*	Forward: GACATGTTTTCTGACGGCAAC Reverse: AAGTCCAATGTCCAGCCC Probe: [FAM]CTGGCAAAGTAGAAAAGGGCGACAAC [BH1]
*ACTB*	Forward: GCGAGAAGATGACCAGGATC Reverse: CCAGTGGTACGGCCAGAGG Probe: [HEX]CCAGCCATGTACGTTGCTATCCAGGC[BH2]

### Statistical analysis

All statistical analyses were performed using GraphPad Prism 5. For the RTCA dataset, one-way ANOVA followed by Tukey’s *post hoc* test was applied. For the qRT-PCR dataset, the Kruskal–Wallis test followed by Dunn’s multiple comparisons test was used. For the SEM dataset, the Mann–Whitney test was applied. All experiments, except SEM analysis, were performed in biological triplicates. Statistical significance was defined as p < 0.05; significance levels in the figures are indicated as *p* < 0.05, *p* < 0.005, and *p* < 0.001.

### Ethics statement

Peripheral blood mononuclear cells were obtained from healthy donors. The participants provided written informed consent to participate in this study. The study was approved by the Local Ethics Committee of Kazan (Volga Region) Federal University (protocol No. 27, dated December 28, 2020). For each experiment, cells from three independent donors (male, 20–30 years old) were used.

## Results

### Ultrasonication generates nanoscale vesicles from T and CAR-T cells

**Figure 1:**
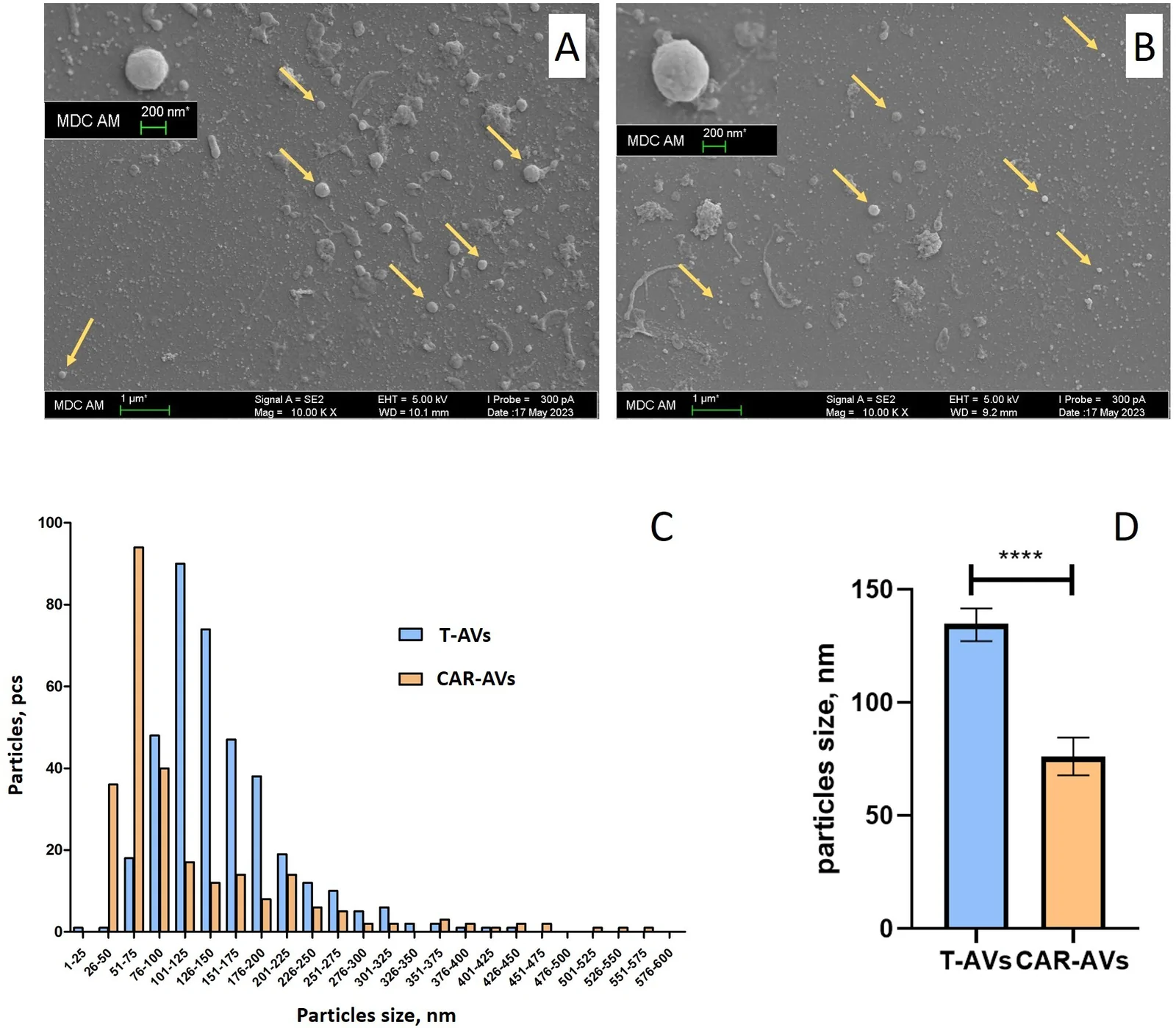
Morphology and size distribution of artificial vesicles generated from T cells and CAR-T cells. **(A, B)** Representative scanning electron microscopy (SEM) images of artificial vesicles (AVs) produced by ultrasonication of unmodified T cells **(A)** and CAR-T cells **(B)**. Yellow arrows indicate individual vesicular particles; insets show higher-magnification views of representative vesicles. Scale bars: 1 µm (main panels) and 200 nm (insets). **(C)** Size distribution of T cell–derived vesicles (T-AVs, blue) and CAR-T cell–derived vesicles (CAR-AVs, orange) determined from SEM micrographs. Vesicle diameters were measured using ImageJ software from 16 independent images per condition (T-AVs, n = 376 vesicles; CAR-AVs, n = 263 vesicles). **(D)** Comparison of vesicle size medians ± 95% confidence interval (CI). Statistical significance was assessed using the Mann–Whitney test; significance level is indicated as p < 0.001 (****). The data demonstrate reproducible generation of nanoscale vesicles within the expected extracellular vesicle size range for both cell types.

Ultrasonication of unmodified T cells and CAR-T cells produced abundant membrane-enclosed particles that were readily visualized by scanning electron microscopy (SEM) (**Figures 1A, B**). Quantitative morphometric analysis of 16 micrographs per condition identified 376 individual T cell–derived vesicles (T-AVs) and 263 CAR-T–derived vesicles (CAR-AVs) (**Figure 1C**). Under identical preparation and isolation conditions, CAR-AVs exhibited a modest leftward shift in the median size distribution relative to T-AVs, which was nevertheless statistically significant (**Figure 1D**). The predominant CAR-AV population ranged between 51–75 nm, extending up to approximately 600 nm, whereas T-AVs were enriched in the 75–200 nm range and rarely exceeded 450 nm in diameter. The mean ± SD diameters were 132 ± 76 nm for CAR-AVs and 149 ± 63 nm for T-AVs.

These size distributions confirm that the applied ultrasonication and 15,000 × g enrichment protocol reproducibly generates vesicles within the expected extracellular vesicle (EV) size range for both cell types. The consistent, although moderate, size reduction observed in CAR-AVs suggests that CAR expression and/or the activation status of donor cells may subtly influence membrane fragmentation under ultrasonic stress. Because vesicle formation in this system is mechanically induced, the applied ultrasonic energy is likely to affect both particle size distribution and retention of membrane-associated proteins: stronger sonication may favor the formation of smaller fragments at the expense of membrane protein preservation, whereas milder conditions may increase the proportion of larger membrane fragments and reduce overall vesicle yield. Importantly, this observation is presented descriptively and does not imply mechanistic differences in vesicle biogenesis.

### CAR is efficiently incorporated into CAR-T–derived vesicles

**Figure 2:**
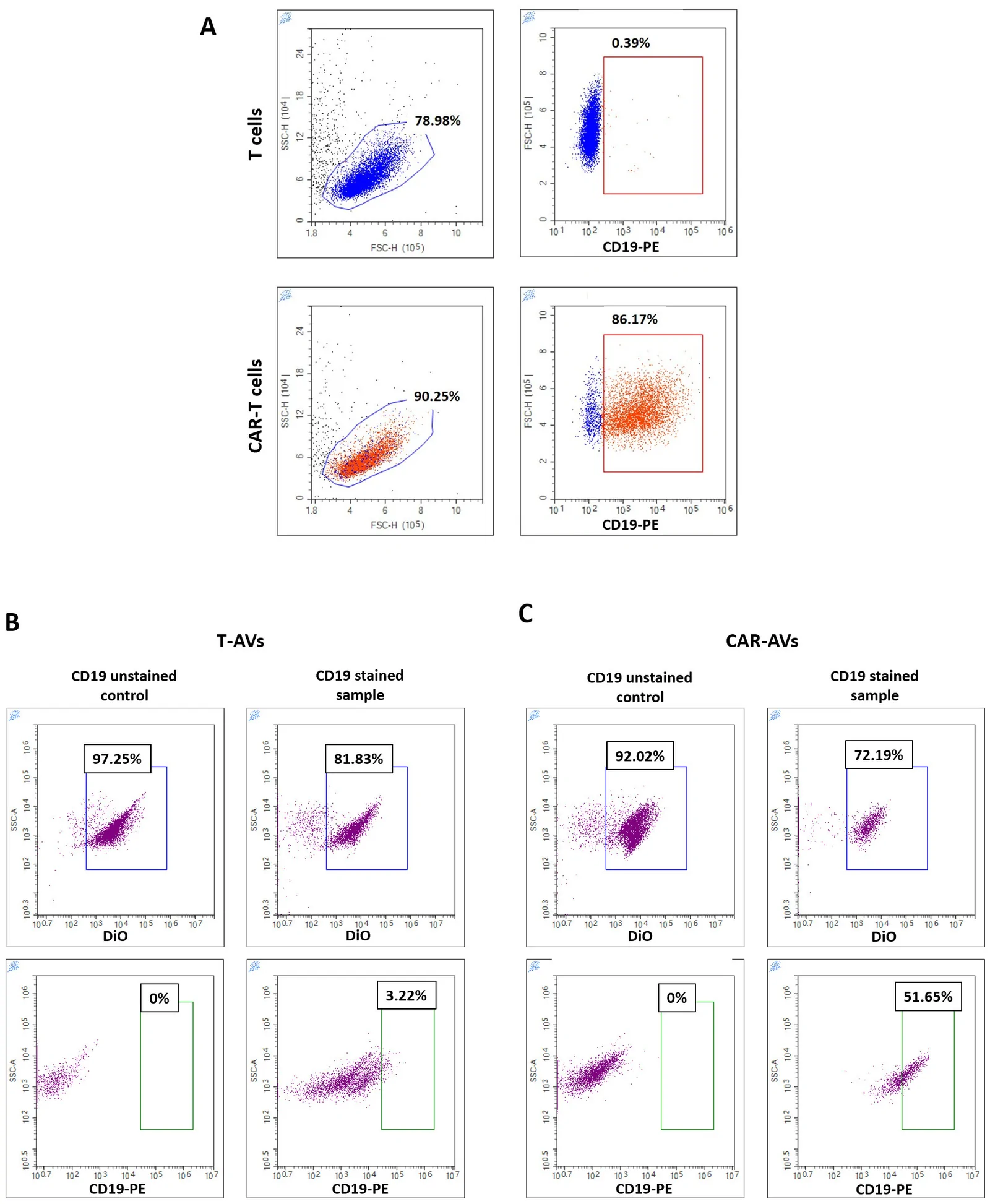
Flow cytometric characterization of CAR expression in parental T cells and derived artificial vesicles. **(A)** Representative flow cytometry plots showing surface CAR expression in untransduced T cells (top) and CAR-T cells (bottom) after staining with recombinant CD19–PE. Cell populations were first gated using forward and side scatter (FSC/SSC) to exclude debris, and CD19–PE–positive events were quantified to determine the proportion of CAR-expressing cells. **(B)** Representative flow cytometric analysis of artificial vesicles (AVs) generated from unmodified T cells (T-AVs). Vesicles were first identified by staining with the lipophilic membrane dye DiO to define vesicle-sized events. Subsequent staining with CD19–PE was used to assess nonspecific background binding within the DiO^+^ vesicle population. **(C)** Corresponding analysis of vesicles derived from CAR-T cells (CAR-AVs) processed under identical conditions. DiO^+^ events were gated as vesicles, and within this population CD19–PE^+^ events represent vesicles displaying CAR on their surface. A total of 10,000 events were acquired for each sample.

To determine whether CAR molecules are retained on ultrasonication-derived vesicles, flow cytometric analysis was performed using DiO membrane labeling to identify vesicular events and recombinant CD19–PE to probe surface CAR (**Figures 2B, C**). In CAR-AV preparations, 52.7 ± 20.5% of DiO^+^ events stained positively for CD19–PE, indicating that approximately half of the analyzed vesicles displayed detectable CAR on their surface. In contrast, control T-AV samples exhibited only low CD19–PE reactivity (8.3 ± 6.1%), consistent with nonspecific background staining. Analysis of the parental CAR-T cells from the same donor batch showed 64.2 ± 14.4% CD19–PE^+^ cells (**Figure 2A**), confirming efficient CAR expression in the original cell population.

Taken together, these results indicate that CAR molecules are effectively incorporated into a substantial fraction of vesicles generated from CAR-T cells by ultrasonication. The low but measurable CD19–PE signal observed in T-AV controls was interpreted as background staining and was taken into account in subsequent functional analyses.

### CAR-AVs selectively suppress proliferation of antigen-positive tumor cells

**Figure 3:**
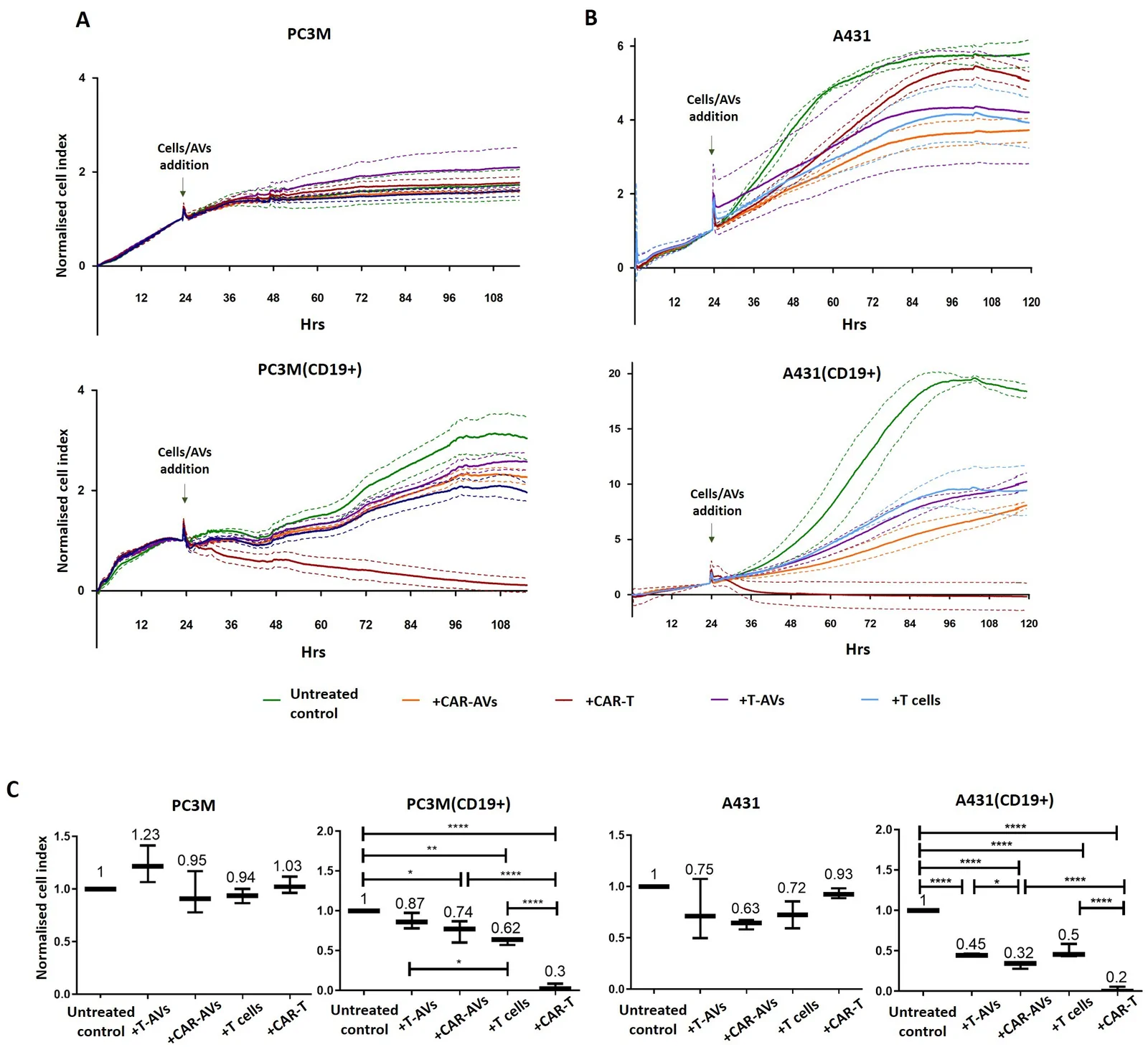
Real-time assessment of tumor cell proliferation in response to T cell– and CAR-T cell–derived artificial vesicles. **(A, B)** Representative xCELLigence real-time cell analysis (RTCA) profiles showing normalized cell index (CI) dynamics for PC3M and PC3M(CD19^+^) cell lines **(A)** and A431 and A431(CD19^+^) cell lines **(B)** following treatment with artificial vesicles (AVs) or parental cells. Tumor cells were treated with vesicles derived from unmodified T cells (T-AVs) or CAR-T cells (CAR-AVs) at a total protein concentration of 15 µg/mL, or co-cultured with live T cells or CAR-T cells at an effector-to-target ratio of 1:1. The vertical arrow indicates the time of treatment addition (24 h). Solid lines represent mean CI values, and dotted lines indicate standard deviations from three independent experiments. **(C)** Quantitative comparison of normalized CI values measured at 94 h for PC3M and PC3M(CD19^+^) cultures and at 72 h for A431 and A431(CD19^+^) cultures. Bars represent median values, with whiskers indicating minimum and maximum values (n = 3). Statistical analysis was performed using one-way ANOVA followed by Tukey’s *post hoc* test; significance levels are indicated as p < 0.05 (*), p < 0.005 (**), and p < 0.001 (****).

The cytostatic and cytotoxic activity of AVs was next evaluated using real-time cell analysis (RTCA) in antigen-negative and CD19-expressing variants of two human solid-tumor cell lines, PC3M and A431. Stable CD19 expression in the engineered lines was confirmed by flow cytometry (**Supplementary Figure 1**). AVs were applied at a protein-normalized concentration of 15 µg/mL, while live T cells and CAR-T cells were included as cellular benchmarks (**Figures 3A, B**).

In PC3M cultures, AV exposure had minimal impact on antigen-negative cells. In contrast, PC3M(CD19^+^) cells displayed a clear reduction in normalized cell index (CI) at 94 h following treatment with CAR-AVs, with a smaller decrease observed after exposure to T-AVs (**Figure 3C**). A more pronounced effect was observed in the A431 model. A431(CD19^+^) cells showed a marked and sustained decline in CI after treatment with CAR-AVs and, to a lesser extent, T-AVs, whereas antigen-negative A431 cells exhibited only minor reductions in proliferation. The endpoint time points – 94 h for PC3M and 72 h for A431 – were selected to coincide with the maximal CI values in untreated controls, ensuring that the observed differences reflected treatment-induced suppression rather than variation in baseline growth kinetics.

Taken together, these findings demonstrate that CAR-AVs preferentially suppress the proliferation of antigen-expressing tumor cells, consistent with CAR-mediated target recognition. The partial activity observed with T-AVs, together with the modest effects detected in antigen-negative cells, suggests the presence of antigen-independent cytotoxic mechanisms that are further explored in subsequent analyses.

### CAR-AV exposure upregulates apoptosis-associated transcripts in target cells

To determine whether the observed growth inhibition was associated with activation of apoptotic signaling pathways, transcript levels of *TP53*, *MDM2*, *CDKN1A (p21)*, *BAX*, and *BBC3 (PUMA)* were quantified after 72 h of co-incubation of tumor cells with T-AVs or CAR-AVs (15 µg/mL total protein).

**Figure 4:**
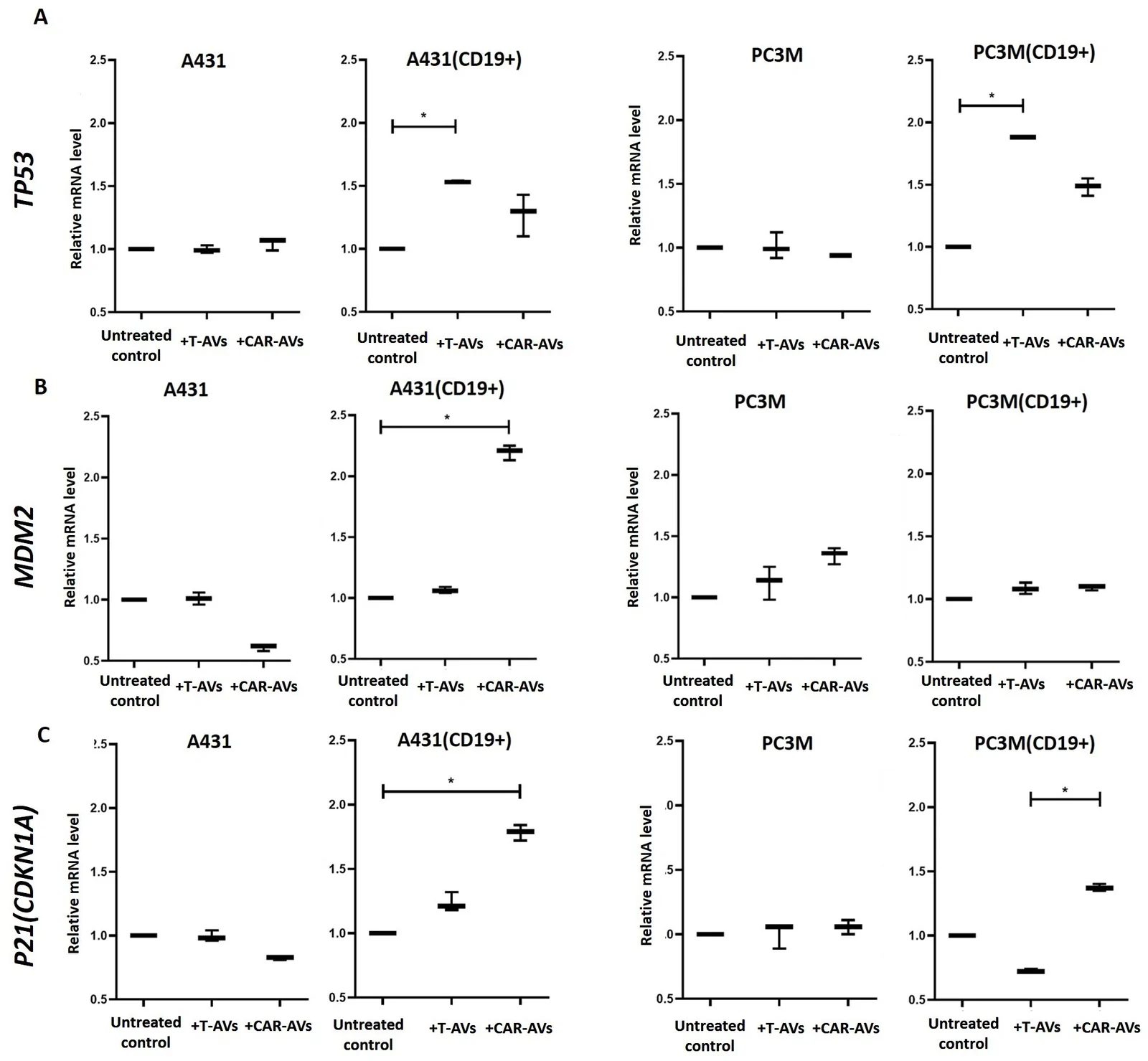
Transcriptional modulation of apoptosis-associated genes in tumor cells treated with T cell– and CAR-T cell–derived artificial vesicles. Relative mRNA expression of **(A)** *TP53*, **(B)** *MDM2*, and **(C)** *CDKN1A (p21)* in A431, A431(CD19^+^), PC3M, and PC3M(CD19^+^) cells following treatment with artificial vesicles derived from unmodified T cells (T-AVs) or CAR-T cells (CAR-AVs). Expression levels were normalized to the housekeeping gene *ACTB* and are shown relative to untreated controls. Bars represent median values, and whiskers indicate minimum and maximum values from three independent experiments (n = 3). Statistical significance was determined using the Kruskal–Wallis test followed by Dunn’s multiple comparisons test; significance thresholds are indicated as p < 0.05 (*).

In antigen-negative A431 and PC3M cells, *TP53* expression remained largely unchanged, with only minor increases detected in their CD19^+^ counterparts (**Figure 4A**). In contrast, several downstream targets associated with p53 signaling displayed more pronounced modulation. In A431(CD19^+^) cells, treatment with CAR-AVs induced a strong upregulation of *MDM2*, whereas antigen-negative A431 cells showed a decrease under the same conditions. No significant changes in *MDM2* expression were observed in the PC3M background (**Figure 4B**).

Similarly, *CDKN1A (p21)* expression increased in both A431(CD19^+^) and PC3M(CD19^+^) cells following exposure to CAR-AVs, with a smaller increase observed in A431(CD19^+^) cells treated with T-AVs. In contrast, antigen-negative cells exhibited either negligible changes or slight reductions in *CDKN1A (p21)* expression (**Figure 4C**).

**Figure 5:**
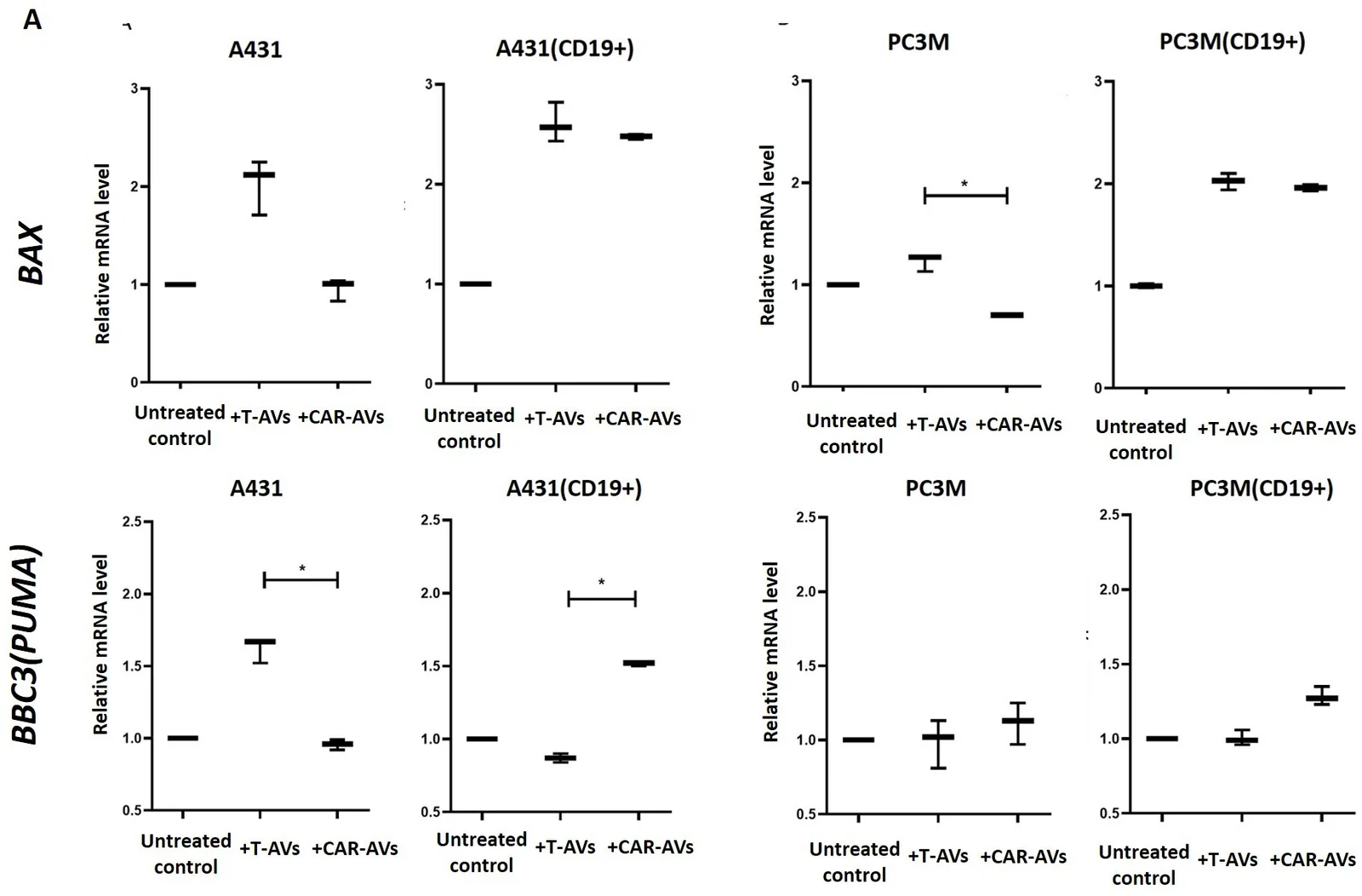
Induction of pro-apoptotic gene expression in tumor cells treated with T cell– and CAR-T cell–derived artificial vesicles. Relative mRNA expression of **(A)** *BAX* and **(B)** *BBC3 (PUMA)* in A431, A431(CD19^+^), PC3M, and PC3M(CD19^+^) cells following treatment with artificial vesicles derived from unmodified T cells (T-AVs) or CAR-T cells (CAR-AVs). Gene expression levels were normalized to the housekeeping gene *ACTB* and are presented relative to untreated controls. Bars represent median values, and whiskers indicate minimum and maximum values from three independent experiments (n = 3). Statistical significance was determined using the Kruskal–Wallis test followed by Dunn’s multiple comparisons test; significance thresholds are indicated as p < 0.05 (*).

For the pro-apoptotic effector genes, *BAX* transcripts were elevated in antigen-positive cells following treatment with either vesicle type (**Figure 5A**). Similarly, *BBC3 (PUMA)* expression increased in A431(CD19^+^) and PC3M(CD19^+^) cells after exposure to CAR-AVs and was also upregulated in A431 cells treated with T-AVs (**Figure 5B**). These transcriptional changes further support activation of apoptosis-associated signaling pathways in antigen-positive tumor cells following vesicle treatment.

Overall, these transcriptional profiles are consistent with the functional effects observed in RTCA assays, indicating that CAR-AVs, and to a lesser extent T-AVs, preferentially activate apoptosis-associated signaling pathways in antigen-expressing tumor cells. The comparatively modest responses detected in antigen-negative cell lines suggest the presence of additional cytotoxic components within the vesicle preparations that act independently of CAR-mediated recognition. Together, these results support a predominantly antigen-dependent activity of CAR-AVs while indicating a secondary antigen-independent component that warrants further mechanistic investigation.

## Discussion

This study addresses a central question in the development of cell-free derivatives of CAR-T therapy: whether vesicles generated from CAR-T cells can retain antigen-specific activity while eliciting measurable antitumor effects. Using a CD19-directed model, we demonstrate that ultrasonication of CAR-T cells produces vesicles (CAR-AVs) in which a substantial proportion display functional CAR on the surface, as verified by recombinant CD19–PE binding. Approximately half of DiO^+^ vesicles were CAR-positive (52.7 ± 20.5%), only modestly below the CAR^+^ fraction in the parental cells (64.2 ± 14.4%). This difference likely reflects the limited membrane area of nanoscale vesicles and lower receptor density (15), rather than systematic loss of CAR during vesicle formation. The minor CD19–PE signal detected in control T-AVs (8.3 ± 6.1%) was interpreted as background and taken into account in subsequent analyses.

At the same time, these flow-cytometric results should be interpreted within the known limitations of conventional cytometry for nanoscale objects. Our reported CAR^+^ fraction reflects detectable DiO^+^ events under the instrument settings used, whereas the smallest particles, particularly those below the practical detection threshold, may be underrepresented. This limitation affects particle-resolved quantification and direct comparisons across preparations. Therefore, complementary approaches such as nanoscale or high-sensitivity flow cytometry, nanoparticle tracking analysis (NTA), and other single-particle methods, including resistive pulse sensing, should be implemented in future studies to better define vesicle number, size distribution, and CAR surface density.

The SEM data confirmed the vesicular morphology of the artificial vesicles; however, the applied sedimentation conditions (15,000 × g) are expected to enrich a mixed population of relatively large vesicles while likely losing exosome-sized particles. In addition, the pellet may contain membrane fragment aggregates. For this reason, more refined purification strategies, such as size-exclusion chromatography, density gradients, or filtration, should be considered in future work. Likewise, particle sizing should be validated by orthogonal approaches, since SEM-derived diameters, even under identical processing conditions, may be influenced by both biological and technical factors, including dehydration, surface deposition, and selection of measurable particles.

The CD19 system was selected as a model antigen for functional testing in order to compare isogenic antigen-negative and antigen-positive variants within the same tumor cell background, thereby minimizing confounding differences between unrelated models. This design allowed us to assess antigen-associated effects in a controlled solid-tumor context. At the next stage of investigation, experiments using naturally CD19^+^ hematologic targets, such as Raji or Nalm-6 cells, and/or CAR constructs directed against clinically relevant solid-tumor antigens, such as PSMA, would further enhance the translational relevance of the platform.

At a total protein concentration of 15 µg mL^–1^, a dose near the lower range typically applied in extracellular vesicle studies (16, 17), CAR-AVs produced a distinct inhibitory effect on the proliferation of antigen-positive tumor cells. Real-time impedance monitoring revealed preferential suppression of PC3M(CD19^+^) and, more prominently, A431(CD19^+^) cells, whereas their antigen-negative counterparts remained largely unaffected, apart from a minor reduction observed in A431. T-AVs also reduced proliferation in antigen-positive cultures, although less consistently and with smaller magnitude. Together, these findings support the coexistence of two mechanistic layers: a CAR-dependent, antigen-restricted effect that mirrors the targeting logic of live CAR-T cells, and an antigen-independent component attributable to T cell–derived cytotoxic mediators, such as death ligands or pro-inflammatory factors, previously reported for T cell EVs (18, 19). Because these factors were not directly quantified here, they are discussed as plausible, literature-supported mechanisms rather than confirmed contributors. Notably, T cell–derived vesicles are known to carry several functionally important proteins, including FasL, perforin, granzyme B, and granulysin, all of which may contribute to cytotoxic activity (20), whereas increasing their specificity through targeting remains an area of ongoing development (21).

Transcriptional analysis of apoptosis-associated genes complements the functional data and supports this dual-mode interpretation. In A431(CD19^+^) cells, CAR-AV treatment elevated *MDM2*, a canonical p53 target, without altering *TP53* transcript levels, suggesting activation of p53 signaling primarily at the post-translational level. In contrast, *MDM2* induction was absent in PC3M-derived models, consistent with prior reports of impaired p53 functionality in this cell line. Although *TP53* mutation status was not experimentally confirmed here, this association is consistent with the known biology of the model. Upregulation of *CDKN1A (p21)* in both CD19^+^ models following CAR-AV exposure, together with real-time evidence of growth arrest, suggests activation of cell-cycle checkpoint pathways. The predominantly antigen-associated increase in *BAX* and *BBC3 (PUMA)*, with *BAX* elevated after exposure to either vesicle type and *PUMA* most strongly induced by CAR-AVs, further supports engagement of stress-responsive and mitochondrial apoptotic pathways. At the same time, these genes can be regulated through both p53-dependent and p53-independent mechanisms, indicating that the observed expression patterns likely reflect convergent cellular stress responses rather than a single linear signaling pathway.

In parallel, the stronger response observed in A431(CD19^+^) relative to PC3M(CD19^+^) is consistent with known differences in p53 pathway integrity and apoptotic competence between these models, although this interpretation should remain conservative and limited to the measured endpoints. More detailed proteomic characterization of AV cargo would also help clarify the mechanisms underlying both antigen-dependent and antigen-independent effects.

Several methodological and translational considerations arise from this work. First, protein-based dose normalization is practical for early-stage *in vitro* studies but does not account for vesicle number, size heterogeneity, or CAR surface density, which complicates potency comparisons across preparations. Second, flow cytometry–based CAR detection provides operational confirmation of surface expression but remains limited by small-particle detection sensitivity and possible nonspecific binding. Third, all experiments were conducted *in vitro*, at a single vesicle dose and fixed time points. These conditions establish feasibility and provide mechanistic direction, but they do not yet define dose-response relationships, pharmacokinetics, biodistribution, or safety margins required for translational development.

Within these boundaries, our results indicate that ultrasonication of CAR-T cells generates extracellular vesicle-sized particles that retain CAR molecules at appreciable frequency, preferentially inhibit proliferation of antigen-positive tumor cells, and induce apoptosis-associated transcriptional programs consistent with targeted cytotoxic stress. A modest nonspecific component is also evident, but it appears quantitatively smaller than the antigen-associated effect. Taken together, these findings position CAR-AVs as a feasible cell-free extension of CAR-T technology and as a practical, scalable platform for further optimization. Rigorous molecular characterization, particle-resolved potency metrics, improved purification, stability assessment, biodistribution studies, *in vivo* efficacy testing, and safety evaluation will be crucial next steps in defining their translational potential.

## Conclusion

Ultrasonication of CAR-T cells produces membrane-bound artificial vesicles (CAR-AVs) that preserve key molecular and functional features of their parental cells. Flow-cytometric analysis using recombinant CD19–PE showed that approximately half of all detected vesicle events displayed CAR, indicating efficient transfer of the chimeric receptor to the vesicle surface. Functionally, at a protein-normalized concentration of 15 µg mL^–1^, CAR-AVs induced a pronounced antigen-associated suppression of tumor cell proliferation, reducing the normalized cell index by approximately 27% in PC3M(CD19^+^) and 68% in A431(CD19^+^) cultures, while exerting minimal effects on antigen-negative counterparts.

These phenotypic effects were accompanied by upregulation of apoptosis-associated transcripts, including *MDM2*, *CDKN1A (p21)*, *BBC3 (PUMA)*, and, in several contexts, *BAX*, consistent with activation of cellular stress and apoptosis-related signaling in antigen-positive targets. A modest antigen-independent component was also observed and is explicitly incorporated into the mechanistic interpretation.

Within the limits of *in vitro* analysis and protein-based dosing, these findings support CAR-AVs as a feasible cell-free extension of CAR-T technology that preserves antigen specificity while inducing apoptosis-associated responses in targeted tumor cells. Future studies should define quantitative dose-response relationships, refine vesicle characterization in terms of particle number and CAR surface density, and clarify the relative contributions of antigen-dependent and bystander mechanisms to the overall biological effect.

## Data Availability

The raw data supporting the conclusions of this article will be made available by the authors, without undue reservation.

## Glossary

ACTB: β-actin
ANOVA: analysis of variance
AVs: artificial vesicles
BAX: BCL2-associated X protein
BCA: bicinchoninic acid (protein assay)
BBC3 (PUMA): BCL2 binding component 3 (p53 upregulated modulator of apoptosis)
CAR: chimeric antigen receptor
CAR-AVs: artificial vesicles obtained from CAR-T cells
CAR-T: chimeric antigen receptor-modified T lymphocytes
CD19: cluster of differentiation 19
CDKN1A (p21): cyclin-dependent kinase inhibitor 1A
CI: cell index
cDNA: complementary DNA
DMEM: Dulbecco’s Modified Eagle Medium
DPBS: Dulbecco’s phosphate-buffered saline
EVs: extracellular vesicles
E:T: effector-to-target ratio
FBS: fetal bovine serum
FSC/SSC: forward scatter/side scatter
IL-2: interleukin-2
MDM2: mouse double minute 2 homolog
MISEV: Minimal Information for Studies of Extracellular Vesicles
MOI: multiplicity of infection
PBMCs: peripheral blood mononuclear cells
PCR: polymerase chain reaction
PE: phycoerythrin
RTCA: real-time cell analysis
RT-qPCR: reverse-transcription quantitative polymerase chain reaction
SD: standard deviation
SEM: scanning electron microscopy
T-AVs: artificial vesicles obtained from unmodified T cells
TME: tumor microenvironment
